# Paving the way toward treatment solutions for CTNNB1 syndrome: a patient organization perspective

**DOI:** 10.1177/26330040251318355

**Published:** 2025-02-12

**Authors:** Špela Miroševič, Shivang Khandelwal, Emily Amerson, Effie Parks, Mariana Parks, Lauren Cochran, Ana González Hernández, Mirela Ferraro, Leszek Lisowski, Andrea Perez-Iturralde, Wendy Chung, Michele H. Jacob, Nina Žakelj, Duško Lainšček, Vida Forstnerič, Petra Sušjan, Matea Maruna, Roman Jerala, Damjan Osredkar

**Affiliations:** CTNNB1 Foundation, The Gene Therapy Research Institute, Dalmatinova ulica 5, Ljubljana 1000, Slovenia; Department for Family Medicine, Faculty of Medicine, University of Ljubljana, Poljanski nasip 58, Ljubljana 1000, Slovenia; CTNNB1 Foundation, The Gene Therapy Research Institute, Ljubljana, Slovenia; CTNNB1 Connect & Cure, Green Village, DE, USA; CTNNB1 Connect & Cure, Green Village, DE, USA; CTNNB1 Connect & Cure, Green Village, DE, USA; CTNNB1 Connect & Cure, Green Village, DE, USA; CTNNB1 Foundation, The Gene Therapy Research Institute, Ljubljana, Slovenia; Asociación CTNNB1 España, Basauri, Spain; CTNNB1 Italia, Via Giovanni Leone, ACRI CS, Italy; Gene Therapy Research Unit, Children’s Medical Research Institute, Faculty of Medicine and Health, University of Sydney, Westmead, NSW, Australia; Gene Therapy Research Unit, Children’s Medical Research Institute, Faculty of Medicine and Health, University of Sydney, Westmead, NSW, Australia; Department of Pediatrics, Boston Children’s Hospital, Harvard Medical School, Boston, MA, USA; School of Medicine, Tufts University, Boston, MA, USA; Department of Pediatric Neurology, University Children’s Hospital, Ljubljana, Slovenia; Department of Synthetic Biology and Immunology, National Institute of Chemistry, Ljubljana, Slovenia; Center for the Technologies of Gene and Cell Therapy, Ljubljana, Slovenia; Department of Synthetic Biology and Immunology, National Institute of Chemistry, Ljubljana, Slovenia; Department of Synthetic Biology and Immunology, National Institute of Chemistry, Ljubljana, Slovenia; Department of Synthetic Biology and Immunology, National Institute of Chemistry, Ljubljana, Slovenia; Department of Synthetic Biology and Immunology, National Institute of Chemistry, Ljubljana, Slovenia; Department of Pediatric Neurology, University Children’s Hospital, University Medical Centre Ljubljana, Ljubljana, Slovenia

**Keywords:** animal disease models, β-catenin, CTNNB1 syndrome, gene therapy, muscle spasticity

## Abstract

The CTNNB1 Connect & Cure and CTNNB1 Foundation, alongside Asociación CTNNB1, CTNNB1 Italia, Association CTNNB1 France, and researchers and clinicians globally are dedicated to finding effective treatments and cures for CTNNB1 syndrome. The syndrome is also characterized by progressive spasticity, which can in some cases cause loss of already achieved motor milestones. Since 2019, they have brought together researchers from different fields and invested in various research efforts to advance the search for treatment solutions for patients with CTNNB1 syndrome. Simons Searchlight serves as an important platform by remotely collecting high-quality, standardized data on the natural history of the disease and making it available to researchers around the world. Conducting genotype–phenotype correlation study and biochemically characterizing the mutations were critical to understand the effects of the patients’ mutations and related molecular function to symptoms. Several induced pluripotent stem cells were generated from patient cells, and preclinical mouse models have provided new insights into the molecular downstream effects of CTNNB1 haploinsufficiency. Multiple therapeutic approaches are in the developing, including small molecule treatments, RNA- and DNA-based therapies, AAV9 gene replacement therapy, which entered the manufacturing phase in November 2023. In this article, we summarize the journey of the CTNNB1 community and its organizations, highlight ongoing and future research projects, and outline the available research resources. The vision for the CTNNB1 community is that in the future several therapeutic options will be available that can be customized to every CTNNB1 patient’s needs.

## Plain language summary

The Role of Patient Organizations in Driving Research and Treatment for CTNNB1 Syndrome

CTNNB1 syndrome is a rare neurodevelopmental disorder that affects the development and maturation of the brain due to changes in the CTNNB1 gene. This gene is responsible for the expression of the beta-catenin protein, which plays an important role in communication between cells and sends signals to other nearby genes that are also important for healthy brain development.

Since 2019, the CTNNB1 Foundation and CTNNB1 Connect & Cure organizations have brought together researchers from different fields and invested in various research efforts to advance the search for treatment solutions for patients with CTNNB1 syndrome. To better understand the disease, natural history and genotype-phenotype correlation studies were conducted with biochemical characterization of the mutations. This was crucial to understand the effects of the patients’ mutations and to link molecular function to symptoms. Several cell and animal models were developed that provided valuable information about the disease.

Importantly, several therapeutic approaches have also been developed and tested on these models. In November 2023, the CTNNB1 Foundation began production for its first gene therapy program and preparations for the clinical trial. With the same enthusiasm, we expect further treatment solutions to enter clinical trials in the next two to three years. Our common goal is to have a combination of therapeutic approaches available in the future that can be tailored to the individual CTNNB1 patient.

## Introduction

Patients and patients’ families are playing an increasingly important role at all stages of therapeutic development. Patient advocacy organizations have a unique opportunity and responsibility to play a major role at different steps of preclinical and clinical development. They help identify and prioritize research areas most relevant to the needs of patients, raise funds to support research, and provide input on clinical trial protocols to ensure they are patient-centered and feasible for patients. They can help strengthen communication between various stakeholders and can support regulatory bodies to provide community and patients’ perspective and advocate to ensure a faster review of new candidate-treatments.

The CTNNB1 Connect & Cure and CTNNB1 Foundation are committed to driving progress in the treatment and cure of CTNNB1 syndrome. Their goal is to build a strong partnership between researchers while providing relentless support to families of affected individuals. We organize international conferences, natural history studies, fundraise to support research and participate in many events to promote, and raise awareness of CTNNB1 syndrome.

To date, our organizations have raised a total of more than $3 million in donations to support various research efforts from multiple international locations. This includes the development and characterization of cellular and animal models of CTNNB1 syndrome and the advancement of various programs to develop treatments for CTNNB1, including small molecule treatment, RNA-based therapy, AAV9 gene replacement therapy (GRT), and DNA modification techniques. In addition, our dedicated researchers have received nearly $1 million in government grants to conduct genotype–phenotype correlation studies and test some of these therapies in preclinical models. Our commitment to exploring multiple avenues of research underscores our unwavering commitment to finding effective treatments for CTNNB1 syndrome.

The CTNNB1 syndrome was first described in 2012 as a genetic cause for cerebral palsy-related disorders.^1^ Since this ground-breaking discovery, the CTNNB1 gene has been included in various genetic testing panels for motor, ocular, and intellectual impairments. The number of patients entering the Facebook community group has continued to increase since 2012 and has reached 500 in 2024. The incidence of CTNNB1 syndrome has been estimated to be 2.6–3.2 in 100,000 births.^2^

The rarity of CTNNB1 and the broad phenotype often delays the diagnosis. Families facing this disease often experience uncertainty about the future of their children due to the paucity of clinical information about adults with the condition.

**Figure 1.**
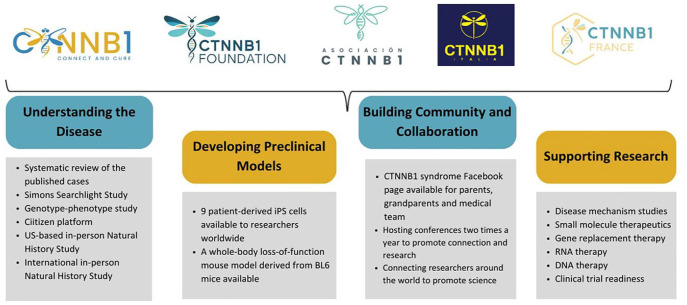
Four pillars demonstrating the CTNNB1 syndrome community mission.

Although we were only recently discovered and have only a modest number of patients, our community has already achieved many milestones. Currently, five patient organizations are working together to serve our community and advance science in the search for treatment solutions for CTNNB1 syndrome. To accomplish the missions shared by all our organizations, we have identified four critical pillars: understanding the underlying disease mechanisms, developing preclinical disease models, building community and collaboration, and supporting research (Figure 1). We present all of these from the patient perspective in this article.

Specifically, we will describe most common CTNNB1 syndrome symptoms with a focus on its association with epilepsy. We will provide a comprehensive overview of our journey as a CTNNB1 community, as well as our current and future research plans. We also present all the resources currently available to researchers worldwide. This patient organization perspective aims to provide a unified framework to promote research for this poorly understood rare disorder.

## About CTNNB1 syndrome

CTNNB1 syndrome is a neurodevelopmental disorder caused by a variety of de novo mutations that result in heterozygous loss-of-function alleles in the *CTNNB1* gene. The disease mechanism is haploinsufficiency. The CTNNB1 gene is located on human chromosome 3, consisting of 16 exons, with exons 2–15 (2346 base pairs) providing the coding sequence for the β-catenin protein.^3^ Because it is an autosomal-dominant disease, a single allelic mutation is sufficient for a pathological phenotype due to insufficient expression of the protein by a single healthy allele. While β-catenin is expressed in virtually all cells and tissues in the body, the most phenotypic consequences likely stem from insufficient levels in the central nervous system (CNS) and skeletal muscles, as reported in animal disease models.^4,5^ Therapies would ideally be targeted to these tissues.

**Figure 2.**
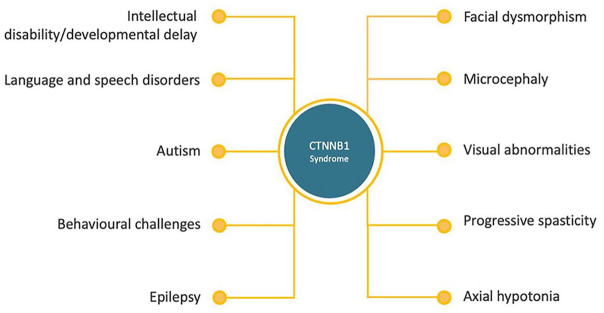
Common features observed in patients with CTNNB1 syndrome.

CTNNB1 syndrome is a severe intellectual disability disorder that drastically impacts both patients and their families throughout their lives. It manifests with symptoms of varying severity, including hypotonia, spasticity, cognitive impairment, language impairment, various behavioral challenges, epilepsy, microcephaly, visual impairment, visual problems including strabismus, and congenital heart defects (Figure 2).^6–9^ Sleep disturbances, including difficulty falling asleep and night-time laughter episodes, have emerged as important sleep-related problems. In addition, increased anxiety and aggressive behavior represent a major challenge in everyday life for patients and their families.^10,11^

### Epilepsy cases in CTNNB1 syndrome

The prevalence of epilepsy in individuals with CTNNB1 syndrome is relatively low and is estimated at 10%–12.5%.^10,11^ A 2022 systematic review by Miroševič et al. reported that three patients had abnormal electroencephalography (﻿EEG) findings characterized by diffuse rapid background activity and epileptiform activity with a tendency to spread.^11^ In addition, a recent study of the natural history of the disease found a prevalence of epilepsy of 12.5%, which included one individual with electrical status epilepticus during slow-wave sleep.^10^

The exact mechanisms by which CTNNB1 mutations cause epilepsy remain unclear. Further research is needed to elucidate the specific molecular interactions affected by CTNNB1 mutations, which could lead to more targeted and effective treatments for epilepsy in this patient group. Understanding these mechanisms is crucial for the development of interventions that not only treat the seizures but also address the general developmental problems associated with CTNNB1 syndrome.

To illustrate, Effie Parks, host of the award-winning podcast Once Upon a Gene, describes her experience with her son’s diagnoses of CTNNB1 and epilepsy:

My 7-year-old son, Ford, has been a fighter since his full-term birth, marked by immediate health challenges due to intrauterine growth restriction (IUGR). His early months were fraught with difficulties, leading to a diagnosis of failure to thrive at three months old. It wasn’t until he was 16 months old, after a whole exome sequencing test, that we received a life-altering diagnosis of CTNNB1 syndrome. Ford’s journey has been unique and perplexing, even to those around us. He exhibited behaviors that, to an untrained eye, seemed unusual but not totally alarming. He had a severe startle response, would often laugh while staring at a corner of the ceiling for hours, and experienced febrile seizures. Yet, the possibility of epilepsy was not immediately considered, partly due to my inexperience as a new parent unaware of what signs to look for, and partly because there weren't enough known cases of CTNNB1 linked to epilepsy at that time. This lack of awareness left Ford vulnerable until a significant physical seizure led to an EEG revealing consistent seizure activity, especially during sleep. The introduction of Keppra brought about behavioral challenges, but it effectively controlled his seizures. This experience underscores the importance of awareness and education about rare diseases like CTNNB1, and the varied ways they can manifest. It’s a reminder of the critical need for shared knowledge and support within the rare epilepsy community.

It is important to note that not every patient with CTNNB1 syndrome develops epilepsy and that the severity and presentation of the syndrome can vary greatly from person to person. Equally important is that epilepsy, although rare, can have a major impact on families and therefore warrants future investigation. As we push forward, it is crucial to deepen our collective knowledge and support systems to improve the lives of those affected by CTNNB1 syndrome and associated epilepsy (https://www.youtube.com/watch?v=nUyp41rvvaM).

## The creation and evolution of CTNNB1 organizations

**Figure 3.**
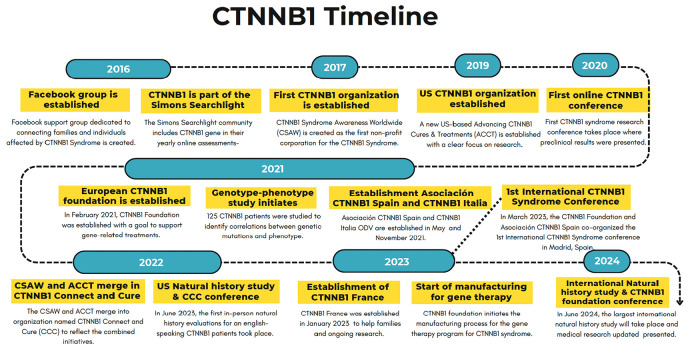
Timeline of major milestones in the CTNNB1 community.

The history of the CTNNB1 organizations and important milestones reached is illustrated in Figure 3. In March 2016, we created a dedicated Facebook support group that marked the first monumental step for the CTNNB1 community. With a growing passion for connecting families and individuals affected by CTNNB1 syndrome, this initiative has since grown into a supportive, family-like community. It is a place where families can feel a sense of belonging, share experiences, provide support, and pass on information. The number of patients included in our studies underscores the collaborative spirit that characterizes the CTNNB1 community and supports the shared mission to find effective treatments and ultimately a cure for this rare disease.^10,11^

In 2017, when there were only 30 known diagnoses worldwide, CTNNB1 syndrome Awareness Worldwide (CSAW) became the first nonprofit corporation created to recognize the rare disease CTNNB1 syndrome. Incorporated as a 501c3 in the United States, the mission of CSAW was to promote awareness of, provide education about, and improve the lives of those with CTNNB1 syndrome. During its first years, a website was created, and the dragonfly was adopted as CTNNB1’s symbol. This symbol was chosen as in many cultures it stands as a symbol of transformation and adaptability, embodying the ability to change and emerge stronger, even in the face of challenges. We felt that this resilient spirit resonates deeply with our CTNNB1 community.

Later, July 25th was established as CTNNB1 Worldwide Awareness Day and a newsletter was launched. The organization’s focus was on connecting CTNNB1 families, both online and in regional meetups, and raising awareness of the issues, concerns, and knowledge of emerging information about the rare disorder.

As CTNNB1 syndrome became more visible, another U.S.-based nonprofit organization, Advancing CTNNB1 Cures and Treatments (ACCT), was established in 2019 to focus on research. Together, CSAW and ACCT hosted online research conferences in 2020 and 2021. Families, clinicians, and researchers from all over the world participated. In 2022, a family meetup was held in Moline, Illinois, where the CTNNB1 community was able to gather face to face for the first time. After working together closely for a couple of years, it was clear that merging organizations would enhance both missions while reducing administrative costs and efforts. Therefore, in 2023, CSAW and ACCT formally merged under the new name CTNNB1 Connect & Cure (CCC). That same year, they held an in-person research conference that included family gatherings and clinical evaluations in addition to informational presentations.

In Europe, the CTNNB1 Foundation was founded on February 5, 2021, after receiving official approval from the Ministry of Health of the Republic of Slovenia. It focuses on the development of advanced gene-related treatments targeting the cause of CTNNB1 disease. The main mission of the CTNNB1 Foundation is to advance and support scientific research that has the potential to lead to the approval of therapies, organize scientific conferences, and support families. In recent years, the CTNNB1 Foundation has published peer-reviewed articles, supported gene-related programs, developed cell and mouse disease models, and organized two scientific conferences. In 2023, a first international CTNNB1 conference brought together 430 researchers, clinicians, CTNNB1 families and other stakeholders from over 17 countries who participated both online and in person. In June 2024, more than 80 families will travel to Slovenia to participate in the “Dragonfly Study,” an international CTNNB1 Natural History Study (NHS). In parallel, presentations focusing on gene therapy, clinical trial readiness, management of the disease, and psychological support will take place.

In recent years, the CTNNB1 community has been enriched with the creation of three additional associations: The Asociación CTNNB1 (Spain) in August 2021, CTNNB1 Italia ODV in November 2021, and CTNNB1 France in January 2023.

Collaboration and communication are vital in rare disease patient organizations like ours. This includes interactions within the CTNNB1 patient community as a whole, within geographical subgroups, between the community and active or potential partners, and with the general public to raise awareness and support fundraising initiatives. Online communication platforms have allowed the CTNNB1 community to find patients, build a support system of affected families, collect patient-centered data, fundraise, heighten awareness, and collaborate all over the world. Communication with other rare disease patient groups is also extremely valuable. Consortiums such as COMBINEDBrain, Rare Epilepsies Network, and Global Genes provide resources and support to build and sustain operations. By communicating and learning from each other in a continually iterative way, rare disease organizations can better optimize their time and energy.

## Together toward treatment solutions for CTNNB1 syndrome

**Figure 4.**
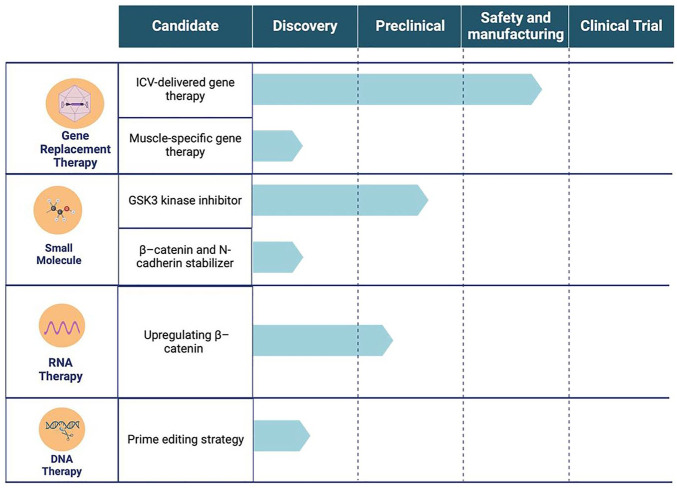
Different treatment options being explored for CTNNB1 syndrome.

As a patient organization, we could not be prouder, as we currently have several treatment strategies in development that target different mutations and severity of symptoms (Figure 4).

GRT involves delivering a functional copy of the CTNNB1 gene into patient cells using viral vectors. Adeno-associated virus (AAV) is the primary vector utilized and continually advanced by researchers for gene therapy due to its nonpathogenic nature and generally nonintegrating properties. Various serotypes with different tissue and cell tropisms have been used in clinical settings to address disease specificity.

Since CTNNB1 deficiency primarily affects cells of the CNS, a functional copy of the CTNNB1 gene will be delivered using the AAV9 serotype. This serotype is also employed in the FDA-approved therapy for spinal muscular atrophy (SMA), Onasmenogene abeparvovec, which is administered intravenously and shown to cross the blood–brain barrier.^12^ Alternative delivery routes, such as intra-cerebroventricular (ICV) administration, provide robust expression across several brain regions and the spinal cord.^13,14^

Two strategies for delivering a functional copy of the CTNNB1 gene are being developed: ICV delivery and muscle-specific delivery. The ICV strategy has already advanced to the manufacturing stage and has shown promising preclinical data. The delivered gene expresses functional β-catenin, thereby restoring normal signaling and cellular functions affected by the disease-causing mutation. The muscle-specific delivery aims to target the muscular phenotypes of the disease, and it is still very early in the development.

Although AAV is the safest tested viral vector, there are still potential risks associated with its use, including immune response with rejection, inflammation, and the theoretical possibility of the inserted gene disrupting other important genes in the patient’s DNA (genotoxicity).^15^ Rigorous safety testing will be conducted before entering clinical trials to address these risks and ensure the therapy’s safety and efficacy.

Small molecule treatment involves two strategies: (a) synthesizing compounds that increase β-catenin levels by inhibiting its degradation, and (b) designing small molecules to stabilize the interaction between β-catenin and N-cadherin. These treatments aim to directly modulate the signaling pathways that are dysfunctional; however, there is a potential for off-target effects where similar pathways are modulated in nontarget cells, possibly leading to adverse effects such as tissue hyperplasia or dysregulation of cellular homeostasis.

RNA-based therapeutic approaches employ the use of antisense oligonucleotide (ASO)-based technology to increase the production of β-catenin from the unaffected allele. By binding to the messenger RNA (mRNA) of the functional allele, ASOs may enhance expression, compensating for the haploinsufficiency. RNA-based therapies can be tuned in terms of dosage and frequency, allowing for treatment adjustments over time based on patient’s response. Advances in oligonucleotide chemistry, including modifications to the backbone and ribose sugar, have significantly enhanced therapeutic properties such as pharmacodynamics, pharmacokinetics, and biodistribution. Notably, extensive chemical modification alone enables tissue delivery of second-generation gapmer ASOs, evident in several approved oligonucleotide therapies.^16^ Administering chemically modified oligonucleotides intrathecally via lumbar puncture has shown to achieve a favorable distribution of therapeutic ASO, such as nusinersen, used in treating SMA, across the CNS.^17^ Risks of oligonucleotide therapy include delivery challenges, such as ensuring that ASOs consistently reach all affected cells, and potential immune responses to the ASOs, which could limit their effectiveness or cause side effects. Meticulous trial design and dosing regimens are needed to develop safe treatment options.

DNA therapy includes the prime editing strategy, an advanced CRISPR-based technique that uses a fusion of Cas9 nickase and reverse transcriptase controlled by a specific pegRNA allowing simultaneous targeting and incorporation of site-specific short deletions, insertions, or point mutations.^18^ This method targets and corrects the DNA sequence directly, without the need for double-stranded breaks, offering a precise and safer approach to potentially cure genetic disorders than methods that cause double-stranded DNA breaks. Screening of several pegRNA variants resulted in identification of candidates with the ability to precisely correct selected *CTNNB1* mutations. While prime editing minimizes unwanted mutations by avoiding double-stranded DNA breaks, its long-term effects are still unclear, and there is still a risk of unintended changes in the genome.

## Understanding the disease: Observational studies and creating CTNNB1 cell and mouse models

To begin exploring different treatment options, our organizations supported basic research to better understand patients’ symptoms, the effects of mutations and the importance of cell and animal models. These studies provide valuable insights into various aspects of the condition, including its clinical manifestations, genotype–phenotype correlations, and the daily challenges faced by patients and their families. Through careful observation and data collection, researchers can identify common symptoms, disease progression patterns, and factors that may influence the severity of the syndrome. These findings are invaluable for both diagnosis and treatment planning, helping clinicians tailor interventions to meet the specific needs of each patient.

Current consensus characterizes CTNNB1 syndrome-associated mutations as loss-of-function^8^; however, it remains unknown whether the loss of β-catenin activity is complete or partial. It also remains to be addressed whether mutations cause loss of β-catenin expression, expression of dysfunctional β-catenin or perhaps even the expression of truncated β-catenin mutants with potentially deleterious effects on the activity of β-catenin expressed from the healthy allele. Understanding the biochemical basis for the genotype–phenotype correlation could allow a more personalized approach to disease management, assist in clinical prediction of the disease severity, and dictate the type of therapeutic intervention in the future. While severe phenotypes associated some mutations may require gene therapy, the milder phenotype associated with other mutations could be managed with less-invasive treatment options.

### Development and initial characterization of CTNNB1 cell and mouse models

The development of CTNNB1 cell and mouse models has been a central aspect of our mission. These models are invaluable and allow researchers to explore the intricate mechanisms of CTNNB1 syndrome and evaluate potential therapeutic strategies.

Testing treatments on CTNNB1 patient cells plays a crucial role in assessing the efficacy of therapeutic interventions, a fundamental step in the development of precise treatments for CTNNB1 syndrome. This approach not only provides insight into the molecular and cellular complications of CTNNB1 syndrome but also opens the door to identifying potential biomarkers or specific cellular changes that correlate with treatment response.

Currently, researchers interested in CTNNB1 syndrome can access cell models with the specific mutations through the Simons Searchlight study (available here: https://base.sfari.org), including pMet328Glufs*24, Ser71Phefs*11, Met174Glnfs*37, Tyr654*, Gly490Alafs*33, Arg95* and Leu452 Glyfs*19. In addition, the CTNNB1 Foundation has made the mutations pMet328Glufs*24, *p.Tyr333Ter* and *IVS6* intron seven available to the research community (contact available here: https://ctnnb1-foundation.org). Importantly, the CTNNB1 Foundation has created a CTNNB1 whole body loss-of-function model derived from C57BL6/J mice. Researchers around the world can access frozen sperm samples from this model upon request (contact available here: https://ctnnb1-foundation.org).

Additionally, researchers worldwide have developed a total of six different animal models, four out of which represent β-catenin deficient, and remaining two represent β-catenin overexpression. In early 2000s, two animal models, one entailing β-catenin conditional knockout (β-catenin is absent only in tissues derived from neural tubes), leading to reduced mass in spinal cord and brain, and a conditional overexpression (β-catenin is overexpressed specifically in tissues derived from neural tubes) causing increased mass in spinal cord and brain were developed.^19^ Then in 2014, a new animal model titled as “Batface” was introduced to the literature by Tucci et al. 2014, harboring Thr653Lys mutation in C-Terminal armadillo repeats of β-catenin. These mice exhibited dysmorphic features and displayed ID like behavior with disrupted motor function and vocalization.^20^ Two years forth, a loss-of-function conditional knockout model having downregulated β-catenin only in Parvalbumin interneurons (PV interneurons) was shown to exhibit autism, ID, behavioral abnormalities, and reduced neuronal activity.^5^ In 2019, as research progressed, a CTNNB1 conditional knockout mice, lacking β-catenin in forebrain excitatory neurons was generated. This model demonstrated severe cognitive impairments, with reduced synaptic adhesion and spine density.^4^ Finally, in 2020, researchers developed a conditional overexpression model with elevated β-catenin levels by selectively knocking out APC, a negative regulator of β-catenin. This model, restricted to forebrain excitatory neurons, exhibited reduced social preference and novelty, alongside increased repetitive behaviors.^21^

In addition to these models, three models that indirectly downregulate the β-catenin levels were introduced in 2009,^22^ 2018,^23^ and 2021.^24^ Another animal model with increased levels of β-catenin was introduced in 2018.^25^ All these models exhibited traits consistent with ID, autism, and schizophrenia along with various molecular features.

### Genotype–phenotype correlation study

In a significant scientific effort to understand the link between genetics and symptoms in CTNNB1 syndrome, an observational study including 125 CTNNB1 patients from all over the world has been completed (NCT04812119).^26^ Its main goal was to interview parents or legal guardians of patients with confirmed CTNNB1 diagnosis and assess them on an extensive questionnaire covering various aspects of the patient’s history, including prenatal and delivery risk factors, current medical issues, and standardized questionnaires on different areas of development and behavior.

The second part of the study was done to investigated the effects of mutations on the function of β-catenin to complement the genotype–phenotype correlation study. Using reporter systems, researchers screened β-catenin constructs bearing mutations for their co-transcriptional activity and structural role. They have determined the expression status of mutants and their tendency to undergo the nonsense-mediated mRNA decay. Additionally, they have determined the potential of expressed mutants to exert negative effects on the function of the β-catenin from the WT allele. In summary, we learned that although the majority of mutations are predicted to be a loss-of-function, in some extremely rare cases, mutations could lead to either a dominant-negative effect or even gain-of-function.^27^

### Natural history study

A crucial component of understanding the natural progression of CTNNB1 syndrome is participation in natural history studies. These studies track the development and experiences of individuals with the condition over time. The information gathered from natural history studies is invaluable in shaping our understanding of the syndrome’s trajectory and guiding clinical decision-making.

The Simons Searchlight study^28^ is an international online research registry oriented toward rare neurogenetic diseases with the aim of documenting their medical, developmental, and behavioral experiences. Information is obtained through online surveys and phone interviews. The Simons Searchlight community encompasses over 150 different genes (see list of genetic conditions www.sfari.org/resource/simons-searchlight/) and more than 20 Copy Number Variations, and CTNNB1 is among the genes under its purview. There are currently 242 CTNNB1 patients registered with Simons Searchlight.

In June 2023, the first in-person NHS evaluations for an English-speaking CTNNB1 patients took place.^10^ Researchers evaluated 32 individuals affected by CTNNB1 syndrome in the study over the course of 2 days. Assessments included motor function (PT), neurological exam, research EEG, and neurocognitive exams. Additionally, 51 blood samples were collected for CTNNB1 biorepositories for research. Deidentified data point and blood samples are available to all accredited researchers through Simons Searchlight.

CTNNB1 syndrome is also tracked through Ciitizen, a digital platform that securely collates medical records of those who participate. Though this database is currently only available for the U.S. participants, it is expected to expand to other countries in the future. This platform gathers retrospective data with minimized burden to patient families by obtaining existing medical records.

Last but not least, the CTNNB1 in-person NHS “Dragonfly Study” is set to begin in June 2024 in Slovenia. This study aims to enroll 80 children or adults of any age and phenotype who have a confirmed genetic diagnosis of CTNNB1 syndrome, along with their primary caregivers. Participants will be asked to attend an annual study visit over a 5-year period. During these visits, both retrospective and prospective clinical data will be collected, including clinical neurological examinations and assessments of motor and cognitive functions, communication, behavior, vision, and sleep. Additionally, brain activity will be recorded through electroencephalography (EEG) and magnetic resonance imaging. Blood tests will also be conducted to reconfirm the underlying mutations in the CTNNB1 gene and to investigate biomarkers of the condition. Caregivers will be invited to complete questionnaires to explore the impact of the condition on their family’s quality of life.

The participation of CTNNB1 syndrome patients in both of these programs not only contributes to a broader understanding of the condition but also allows them to share their unique journeys and challenges with a global network of researchers and families facing similar circumstances. By actively engaging with NHS like Simons Searchlight and Ciitizen, we move closer to addressing the individualized needs of CTNNB1 syndrome patients. This collaborative effort serves as a beacon of hope for better-informed care and the development of tailored therapeutic strategies, ultimately enhancing the quality of life for those affected by this rare genetic condition.

### Future needed studies

A crucial aspect in the early stages of clinical development is the ability to reliably measure an effect that clearly indicates efficacy. Conventional hospital scales used by physiotherapists, psychologists, or neurologists are often not sensitive enough to detect minor effects, making it difficult to achieve clinical significance within a reasonable time frame. For this reason, we are investigating the Syde^®^ device, which is being developed to assess the physical condition of people with movement disorders. The European Medicines Agency has qualified SV95C-a digital outcome calculated with these sensors – as a secondary^29^ then a primay^30^ endpoint in Duchenne muscular dystrophy (DMD). Five of our patients with CTNNB1 syndrome participated in a preliminary study with the Syde^®^ device in February 2024. In addition, 20 CTNNB1 patients will take this device home for a few months to try to obtain disease-specific variables that can help evaluate treatment efficacy. This device will be attached to the children’s limbs to monitor potential clinical outcomes.

In the future, a comprehensive retrospective study to enroll individuals diagnosed with CTNNB1 syndrome needs to be conducted. For rare diseases, especially those that have only recently been discovered and for which little long-term data are available, understanding disease progression is of paramount importance. While retrospective study designs rely on participants’ recollection of past events, people may not remember milestones accurately or their memories may be influenced by their current circumstances or beliefs. Nevertheless, it is important to collect information about when a child has stopped reaching milestones or when they have regressed in their achievements (e.g., walking independently). These data can be of great use to clinicians as it can help to identify potential time windows for enrolling patients into the clinical trial.

## Conclusion

In summary, the central role of patient organizations has been instrumental in advancing our understanding of CTNNB1 syndrome and supporting research to develop effective therapies to improve patients’ quality of life. While it is unfortunate that rare diseases such as CTNNB1 syndrome often rely heavily on patient organizations, it is also a testament to the strength and resilience of parent-led rare disease organizations and communities that have taken the lead in driving change and innovation.

We are deeply grateful to all researchers and their organizations for their tireless commitment to researching our gene and working with our community to improve outcomes for children affected by CTNNB1 syndrome. Through genotype–phenotype correlation studies, natural history studies, and the creation of various cell and animal models, our understanding of CTNNB1 syndrome and its associated symptoms and behaviors has deepened substantially. These studies and models have provided invaluable insights into the role of CTNNB1 in brain development, motor function, cognitive impairment, and behavioral abnormalities. They have also enabled researchers to identify potential therapeutic targets and explore treatment strategies for this rare disorder.

In addition, the availability of patient-derived cell models and mouse models of a CTNNB1 loss-of-function model has opened exciting avenues for collaboration and research in the field of CTNNB1 syndrome. However, it is important to recognize that animal models have their limitations and may not fully reflect the subtleties of CTNNB1 syndrome in humans. Therefore, it is imperative that further research and human clinical trials are conducted to validate the findings from the animal models and translate them into effective treatments for people with CTNNB1 syndrome.

Through the concerted efforts of patient organizations, dedicated researchers, and the CTNNB1 community, we aim to shine a brighter light on CTNNB1 syndrome and show potential researchers that our community has freely available resources, a wealth of knowledge, and a strong, united front in the pursuit of clinical readiness and improved outcomes for people living with this rare disease. These efforts, combined with ongoing observational studies and international collaborations, provide hope for the future in developing personalized and effective therapies for CTNNB1 syndrome patients.

## Declarations

**Ethics approval and consent to participate:** Not applicable.

**Consent for publication:** Not applicable.

**Availability of data and materials:** Not applicable.
